# Factors affecting cumulative live birth rate after the 1st oocyte retrieved in polycystic ovary syndrome patients in women during IVF/ICSI-ET

**DOI:** 10.1186/s13048-023-01290-3

**Published:** 2023-10-13

**Authors:** You Li, Leizhen Xia, Zengming Li, Ziyu Zhang, Ru Jiang

**Affiliations:** 1 Reproductive Medicine Center, Jiangxi Maternal and Child Health Hospital, Nanchang, Jiangxi P. R. China; 2 The Subcenter of National Clinical Research Center for Obstetrics and Gynecology, Jiangxi Maternal and Child Health Hospital, Nanchang, Jiangxi Province China; 3 Clinical Research Center for Obstetrics and Gynecology of Jiangxi province, Jiangxi Maternal and Child Health Hospital, Nanchang, Jiangxi P. R. China; 4 Department of pathology, Jiangxi Maternal and Child Health Hospital, Nanchang, Jiangxi P. R. China; 5 Department of gynecology and obstetrics, The First Affiliated Hospital of Nanchang University, Nanchang, Jiangxi P. R. China

**Keywords:** Cumulative live birth, The 1st oocyte, IVF-ET, PCOS, Cox proportional risk regression model

## Abstract

**Background:**

The factors affecting the cumulative live birth rate (CLBR) of PCOS (Polycystic ovary syndrom) patients who received in vitro fertilization/intracytoplasmic sperm injection-embryo transfer (IVF/ICSI-ET) needs more research for a better outcome.

**Methods:**

Here we carried out a retrospective analysis of 1380 PCOS patients who received IVF/ICSI-ET for the first time from January 2014 to December 2016. We divided them into cumulative live birth group (group A) and non-cumulative live birth group (group B) according to whether there were live births.

**Results:**

The conservative cumulative live birth rate was 63.48%. There were 876 cumulative live births (group A) and 504 non-cumulative live births (group B) according to whether the patients had live births or not. Competition analysis showed that duration of infertility, primary/secondary type of infertility, stimulation protocols, starting dose of gonadotrophins and oocyte retrieved numbers were significantly correlated with CLBR. The Cox proportional risk regression model of PCOS patients showed that stimulation protocols had a significant impact on CLBR. Patients in the GnRH (Gonadotropin-releasing hormone)-antagonist protocol group and the mild stimulation protocol had lower CLBR than those in the prolonged GnRH-agonist protocol, which was statistically significant. PCOS patients with the starting dose of gonadotrophins greater than 112.5u had lower CLBR than those with less than 100u, which was statistically significant. Women with 11–15 oocytes and 16–20 oocytes had higher CLBR than women with 1–9 oocytes, which was statistically significant.

**Conclusions:**

When we used Prolonged GnRH-agonist protocol, or the first starting dose of gonadotrophins was 100u-112.5u, or the number of oocytes obtained was 11–15 and 16–20, the CLBR of PCOS patients increased significantly after the 1st oocyte collection.

## Background

Polycystic ovary syndrome (PCOS) is a prevalent endocrine disorder among women, impacting approximately 10% of women of reproductive age [1]. In cases where patients with PCOS experience infertility despite undergoing multiple ovulation-induced treatments, they are frequently recommended to undergo in vitro fertilization (IVF) or intracytoplasmic sperm microinjection (ICSI) [2]. However, PCOS patients frequently experience overweight or obesity, hormone metabolism disturbances, insulin resistance (IR), and various endocrine and metabolic irregularities during the IVF/ICSI treatment process. These factors have the potential to adversely impact oocyte quality, early embryo development, and endometrial receptivity [3]. Hence, the demographic characteristics of individuals with polycystic ovary syndrome (PCOS), including age, body mass index (BMI), number of antral follicles (AFC), duration of infertility, primary or secondary infertility status, and basic hormone levels, alongside the clinical interventions employed (such as treatment protocols, gonadotropin usage, sex hormone levels on human chorionic gonadotropin (HCG) trigger day, endometrial thickness on HCG trigger day, number of retrieved oocytes, and insemination method), exert a significant influence on the outcome of in vitro fertilization (IVF) or intracytoplasmic sperm injection (ICSI). For instance, it has been observed that women below the age of 35 exhibit a higher live birth rate (LBR) compared to women aged 35 and above in IVF-ET treatment [4]. Additionally, women with a BMI exceeding 28 kg/m2 have been found to have a lower LBR [5]. Furthermore, the LBR of patients is influenced by treatment protocols and the administration of gonadotropin [6–8]. Notably, patients who had 6–15 oocytes retrieved displayed the highest LBR and experienced fewer complications [9]. Despite extensive research on this matter, the key factors associated with it remain unclear [6, 8, 10, 11]. Consequently, the present study was conducted to examine the data of patients with PCOS who underwent IVF/ICSI-ET, with the aim of identifying the pertinent factors that influence the outcomes of IVF/ICSI-ET in PCOS patients. Accordingly, the cumulative live birth rate (CLBR), encompassing the combined live birth rate of both fresh and thawing cycles subsequent to the initial oocyte collection, was selected as the ultimate evaluative measure [12, 13]. In this study, we retrospectively analyzed the clinical data of PCOS patients, and divided them into cumulative live birth group (group A) and non-cumulative live birth group (group B) according to whether there were live births.

## Materials and methods

### Patients selection

This study was a retrospective analysis of PCOS patients who received in IVF/ICSI for the first time from January 2014 to December 2016 at the Reproductive Medicine Center of Jiangxi Maternal and Child Health Hospital, P.R. China. The data of fresh and thawed the embryos transfer cycle after 1st oocyte retrieved were collected and were followed for 2–4 years until December 2018. PCOS patients were diagnosed based on the Rotterdam criteria [14], including oligo-ovulation or anovulation and polycystic ovary (PCO). Exclusion criteria included unilateral ovariectomy, recurrent spontaneous abortion (defined as the loss of three previous spontaneous pregnancies), congenital or acquired uterine malformations, abnormal karyotype analysis, and the exclusion of endometriosis, uterine fibroids, adenomyosis, severe hyperprolactinemia and thyroid disease history. At the same time, we also excluded the cycles in which live births have not yet been obtained but have frozen embryos remaining, and clinical pregnancies that have not yet been delivered (as shown in the flow chart below). According to the cumulative live births of PCOS patients after 1st oocyte collection, we divided them into cumulative live births group (group A) and non-cumulative live births group (group B).

**Figure:**
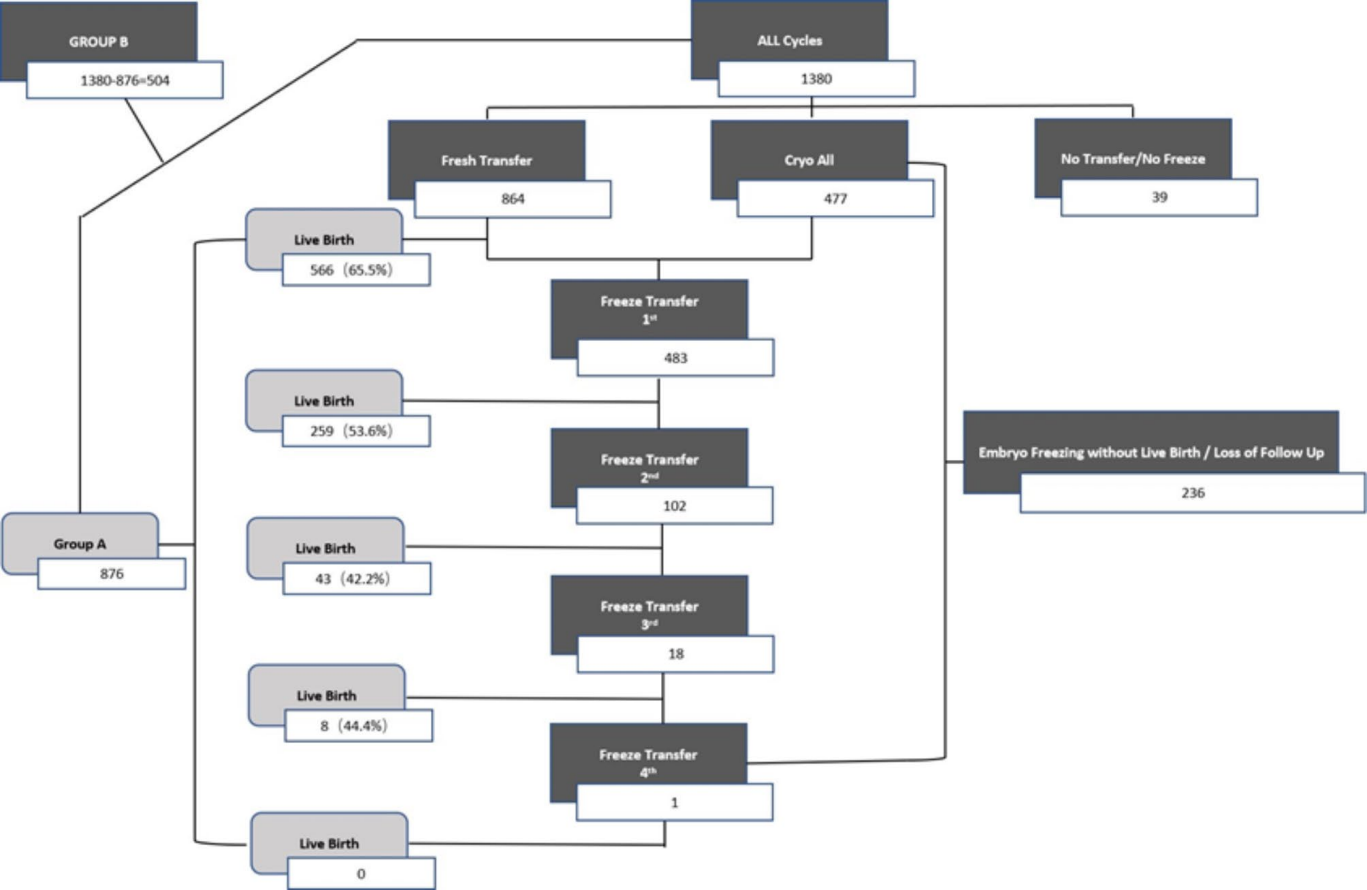
The flow chart of number of frozen embryos or fresh embryo transfers

### Blood sampling and sex hormone measurement

Blood samples were collected on the third day of the menstrual cycle and on the day of HCG injection. The serum was used for the quantitative determination of sex hormone (follicle-stimulating hormone (FSH), luteinizing hormone (LH), estradiol (E_2_), prolactin (PRL) and testosterone (TES)) level by chemiluminescent enzyme immunoassay using Automated Enzyme Immunoassay Analyzer (AIA-2000ST, TOSOH CORPORATION).

### Treatment protocols and embryos transfer

#### Prolonged GnRH-agonist protocol

GnRH-a (3.75 mg) was used in the second or three day of menstrual cycle in prolonged GnRH-agonist protocol. Gonadotrophin stimulation were started after 28 or 38 days following the criteria: no ovarian cysts > 8 mm, E_2_ < 50pg/ml, FSH < 5 u/L, LH < 5 u/L. Initial, patients received 75-150u/d of gonadotrophins according to the patient’s age, BMI, serum basal FSH levels, LH levels, E_2_ levels and antral follicle count.

### GnRH-agonist protocol

All subjects received oral contraceptive pills (OCP) for 21 days starting on menstrual day 5 in the cycle prior to the treatment cycle in GnRH-agonist protocol. Subcutaneous injection of triptorelin 0.1 mg was given on day 21 of OCP administration and continued until the triggering day. gonadotrophins injection with a dose of 75–150 u daily was started on the next menstrual on day 3.

### GnRH-antagonist protocol

On day 2 or 3 of the menstrual cycle, when the follicular diameter of the patients was less than 8 mm and the blood E_2_ was less than 50 pg / ml, gonadotrophins injection with a dose of 75-150u daily was started. When the dominant follicle reached a diameter of 12 mm or the level of estradiol were > 200 pg/mL, GnRH antagonist 0.25 mg was given daily afterwards. Treatment with antagonist and gonadotrophins was continued until the triggering day.

### Mild stimulation protocol

Oral administration of letrozole 2.5 mg/d or clomiphene 50 mg/d on the third day of menstruation, and fifth days of intramuscular injection of gonadotrophins 75-150u/d, according to the growth of follicles, adjust the dose of gonadotrophins to trigger day. GnRh antagonist 0.25 mg-0.5 mg must be added when the LH level begins to rise in mild stimulation. GnRH-a 0.1 mg and HCG 2000u were injected intramuscularly that night in the mild stimulation protocol.

### Oocyte collection and zygote scoring

The time and dose of gonadotrophins were adjusted according to ovarian response as monitored by serum E_2_ levels and vaginal ultrasound. When the dominant follicle was ≥ 19 mm in diameter or at least 3 follicles were ≥ 17.5 mm in diameter and 1/3 follicles larger than 1.4 cm, recombinant human FSH was stopped, and a single injection of 250ug of HCG (Merck-Serono, Darm-stadt, Germany) was administered in the Prolonged GnRH-agonist protocol, GnRH-agonist protocol and GnRH-antagonist protocol. According to E_2_ value, HCG 1000u − 2000u (1000 u/branch, China Li Zhu company) was added. After 36–38 h, the oocytes were taken by transvaginal ultrasound-guided transvaginal puncture. One or two embryos were transferred 3–5 days after oocytes collection under the guidance of abdominal B-ultrasound. After 34–36 h, the oocytes were removed by operation. If the patient used the mild stimulation protocol, moderate to severe OHSS might occur, P level is higher or other situation which were not suitable for transplantation, the transplantation is cancelled and all embryos are frozen after informed consent of both the patient and his wife.

The planned transplant patients received intramuscular injection of progesterone 80 mg daily from the day of oocyte retrieval. After the embryos transfer, they were given intramuscular injection of progesterone 60 mg/d or modified progesterone vaginal agglomerate (Orenorone, Merck) for vaginal route, plus Dydrogesterone tablets (Duphaston, 10 mg/tablets, Solvay pharmaceutical) oral administration, bid. Four weeks later, the clinical pregnancy was confirmed by ultrasound. After the occurrence of fetal heart rate, the luteal support drug gradually decreased.

### Freeze-thaw embryo transfer

Thawed embryo transfer will be carried out in patients with fresh cycle whole embryo cryopreservation or patients with previous embryo cryopreservation without live birth at an optional time. The hormone replacement cycle begins on the 2–4 day of menstrual cycle, oral Estradiol valerate tablets (1 mg/tablets, German Bayer medical care) 4–8 mg/d. The endometrial thickness is monitored by B-mode ultrasound, and the dosage is increased according to the endometrial thickness. When the thickness of endometrium was ≥ 7 mm after 12–16 days, progesterone injection was added for 80 mg/d. Cleavage embryo or blastocyst transfer at 4 or 6 days after injection.

Live birth is defined as the newborns with over 28 weeks of gestation and one of the four vital signs of heartbeat, respiration, umbilical cord pulsation and voluntary muscle contraction after delivery. The cumulative live birth rate is the total probability that the total live birth rate after transferring all the embryos which are from one oocyte retrieved, excluding those who have not yet obtained a live birth and still have frozen embryos, as well as those who have received clinical pregnancy but have not yet obtained a live birth.

### Statistical analysis

The differences between the two groups in general characteristics and clinical treatment data were statistically analyzed. The competitive risk model was used to analyze the single factor of CLBR, and Cox proportional risk regression model was used to evaluate which factors had more influence on CLBR. The characteristics of the live birth patients (group A) and no live birth patients (group B) were compared. For continuous variables, Student’s t-test was used for data with homogeneous variance and the Chi-square tests were applied to detect differences between categorical variables. The P-values correspond to tests for difference between the two groups with a significance level of 5%. Data are presented as mean (SD) or number (%) as relevant. The CLBRs were compared by Gray’s test using a competing risk analysis. Cox proportional hazard model was used to evaluate the relative prognostic significance of female age, duration of infertility, type of infertility, treatment protocols, starting dose of gonadotrophins and oocyte retrieved in relation to CLBR. Interactions between the independent covariates were tested. For age, subjects were categorized into four age groups (< 30, 30–34, 35–38 and > 38 years). They were divided into three group (< 2, 3–4 and > 4 years) according to the years of infertility. They are divided into 3 groups (< 100u, 100u–112.5u and > 112.5u) based on the starting dose of gonadotrophins. The four groups for the number of retrieved oocytes were 1–10, 11–15, 16–20 and > 20. All analyses were conducted using SAS software, version 9.4 (SAS Institute Inc., Cary, NC, USA).

## Results

### Patient characteristics

We enrolled 1380 PCOS patients who underwent the 1st oocyte retrieval cycle. The birth outcomes were followed up for 2–4 years, including fresh and thawed cycles. The conservative cumulative live birth rate was 63.48%. Among them, there were 1087 Prolonged GnRH-agonist protocol, 222 GnRH-agonist protocol, 57 GnRH-antagonist protocol and 14 mild stimulation protocol.

As shown in Table 1, the distribution of infertility years in group A was significantly different from that in group B (*p* = 0.0439). The number and proportion of patients with infertility more than 4 years in the two groups were 298 (35.27%) and 206 (41.78%), respectively. The proportion of primary infertility and secondary infertility in group A was significantly different from that in group B (*p* = 0.0338). The number and corresponding proportion of patients with primary infertility in the two groups were 620 (70.78%) and 329 (65.28%). There was no statistical difference in the results of basic hormones levels (FSH, LH, E_2_, PRL, T), age distribution, BMI and AFC values between the two groups.

**Table 1:** Patient characteristics between two groups

Characteristics	Group A	Group B	t/${{\upchi }}^{2}$	p
Numbers of cycle	876	504		
AGE(Years)				
< 30	679(77.51%)	368(73.02%)		
30–34	173(19.75%)	113(22.42%)		
35–38	21(2.4%)	19(3.77%)		
> 38	3(0.34%)	4(0.79%)	5.3176	0.15
BMI( Kg/m^2^)	22.96 ± 3.48	23.28 ± 3.53	1.67	0.0955
AFC	22.57 ± 5.1	22.28 ± 4.57	0.91	0.3651
Duration of Infertility (Years)				
0~2	204(24.14%)	99(20.08%)		
3~4	343(40.59%)	188(38.13%)		
> 4	298(35.27%)	206(41.78%)	6.2535	0.0439
Type of Infertility				
Primary Infertility	620(70.78%)	329(65.28%)		
Secondary Infertility	256(29.22%)	175(34.72%)	4.5036	0.0338
bFSH	5.85 ± 1.56	5.7 ± 1.46	1.64	0.102
bE2	52.27 ± 156.89	48.51 ± 93.96	0.54	0.5913
bPRL	15.68 ± 8.81	16.5 ± 19.43	0.85	0.3951
bLH	9.82 ± 7.18	9.39 ± 5.88	1.17	0.2411
bT	43.48 ± 18.44	44.38 ± 17.69	0.84	0.4032

Group A: Live Birth; Group B: NO Live Birth;

### Clinical outcomes

In the treatment plan comparison (Table 2), the treatment plan distribution of group A and the ratio of group B were statistically significant (*p* < 0.0001). The number and proportion of prolonged GnRH-agonist protocol cases in the two groups were 729 (83.22%) and 358 (71.03%), respectively. The number and proportion of 112.5u dose groups in the two groups were 398 (45.43%) and 203 (40.28%), respectively. The proportion of 11–15 oocytes and 16–20 oocytes in group A was higher than that in group B (258, 29.45% and 110, 21.83%; 243, 27.74% and 122, 24.21%, *p* < 0.0001), respectively. The number of available embryos in group A was higher than that in group B (4.81 ± 2.91 and 3.41 ± 2.69, *p* < 0.0001). There was no statistical difference in total gonadotrophins, total days of gonadotrophins, LH, P, E_2_ and endometrial thickness between the two groups on HCG day. Furthermore, no statistical difference was found in the proportion of fertilization methods and the number of embryo transfer between the two groups.

**Table 2:** Clinical treatment between two groups

Characteristic	NO Live Birth	Live Birth	t/${{\upchi }}^{2}$	p
Numbers of cycle	876	504		
Treatment Protocols				
Prolonged GnRH-agonist	729(83.22%)	358(71.03%)		
GnRH-agonist	113(12.9%)	109(21.63%)		
GnRH-antagonist	29(3.31%)	28(5.56%)		
Mild Stimulation	5(0.57%)	9(1.79%)	29.7399	< 0.0001
Start Dose of Gonadotrophins(IU)				
< 100	87(9.93%)	50(9.92%)		
100	324(36.99%)	180(35.71%)		
112.5	398(45.43%)	203(40.28%)		
> 112.5	67(7.65%)	71(14.09%)	15.3588	0.0015
Total Dose of (IU)Gonadotrophins	1947.13 ± 1007.42	1934.7 ± 1105.3	0.21	0.8356
Total days of Gonadotrophins	12.95 ± 3.25	12.61 ± 3.27	1.87	0.0617
LH on HCG trigger day (mIU/mL)	1.22 ± 1.18	1.43 ± 1.51	2.53	0.0116
P on HCG trigger day (ng/mL)	0.6 ± 0.81	0.69 ± 0.77	0.64	0.5201
E2 on HCG trigger day (pg/mL)	3253.91 ± 1845.03	3332.21 ± 1922.96	1.49	0.1397
Endometrial thickness on HCG trigger day (mm)	10.69 ± 2.12	10.5 ± 2.49	1.46	0.1433
Oocytes Retrieved				
1~10	150(17.12%)	132(26.19%)		
11~15	258(29.45%)	110(21.83%)		
16~20	243(27.74%)	122(24.21%)		
> 20	225(25.68%)	140(27.78%)	21.8899	< 0.0001
Insemination method				
IVF	736(84.02%)	419(83.13%)		
ICSI	103(11.76%)	59(11.71%)		
IVF + ICSI	37(4.22%)	26(5.16%)	0.6432	0.725
Embryos transferable	4.81 ± 2.91	3.41 ± 2.69	8.84	< 0.0001
Numbers of embryo transferred	1.79 ± 0.4	1.78 ± 0.43	0.49	0.6252

Group A: Live Birth; Group B: NO Live Birth

### Gray’s test

**Fig. 1:**
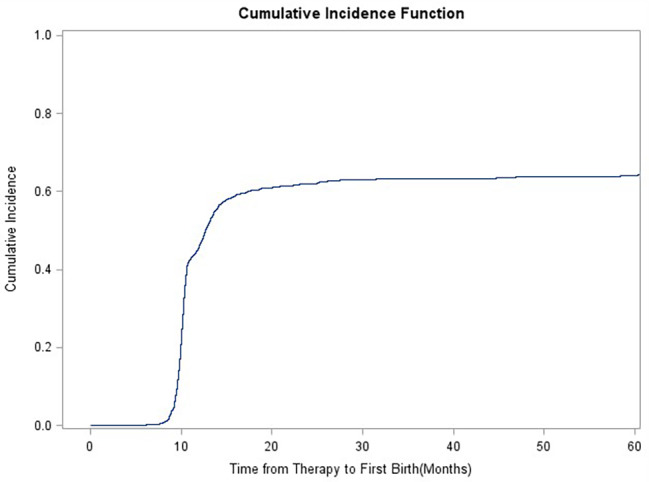
Cumulative live birth rates (CLBRs) in PCOS patients including all first deliveries during 2–4 years follow-up

**Fig. 2:**
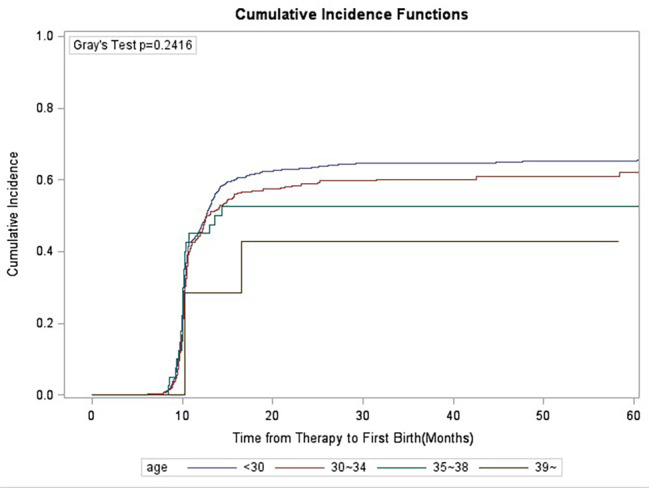
CLBR, stratified for age in three groups < 30 years, 30–34 years, 35–38 years and > 36 years. The distributions were similar (P = 0.2416)

**Fig. 3:**
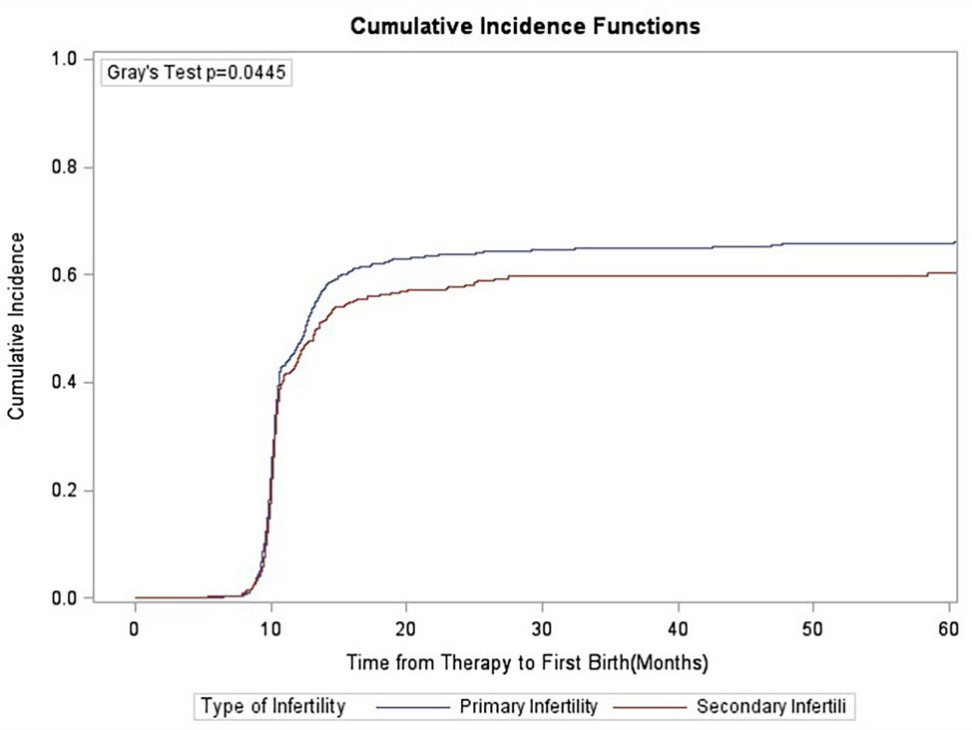
CLBRs in PCOS patients with secondary infertility were significantly higher than the people with Primary infertility (P = 0.0445)

**Fig. 4:**
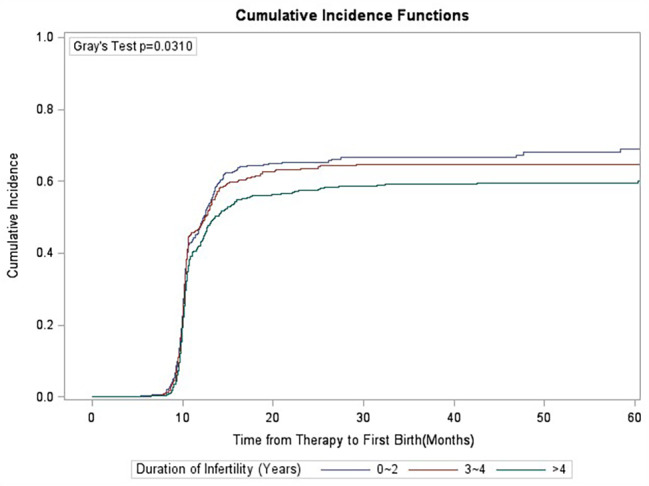
CLBRs, stratified for duration of infertility years in three groups 0–2, 3–4 and ≥ 4. The distribution differed significantly (P = 0.031)

**Fig. 5:**
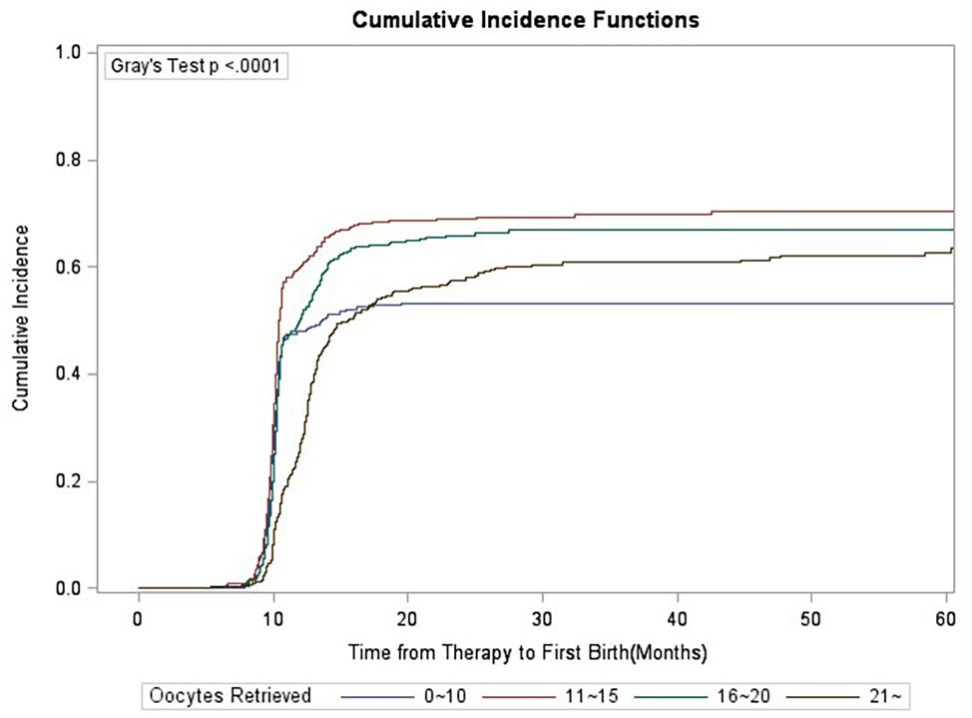
CLBRs, stratified for number of retrieved oocytes in four groups 0–10, 11–15, 16–20 and > 20 oocytes. The distribution differed significantly in the groups (P < 0.0001)

**Fig. 6:**
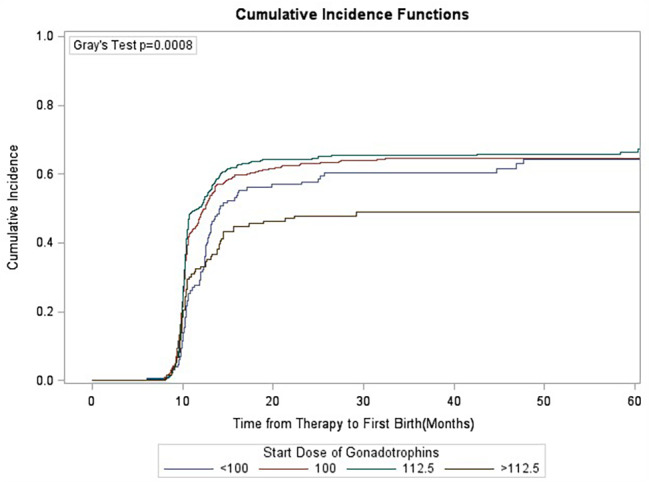
CLBRs, stratified for start dose of gonadotrophins in four groups < 100IU, 100IU, 112.5IU and > 112.5IU. The distribution differed significantly in the groups (P < 0.0008)

**Fig. 7:**
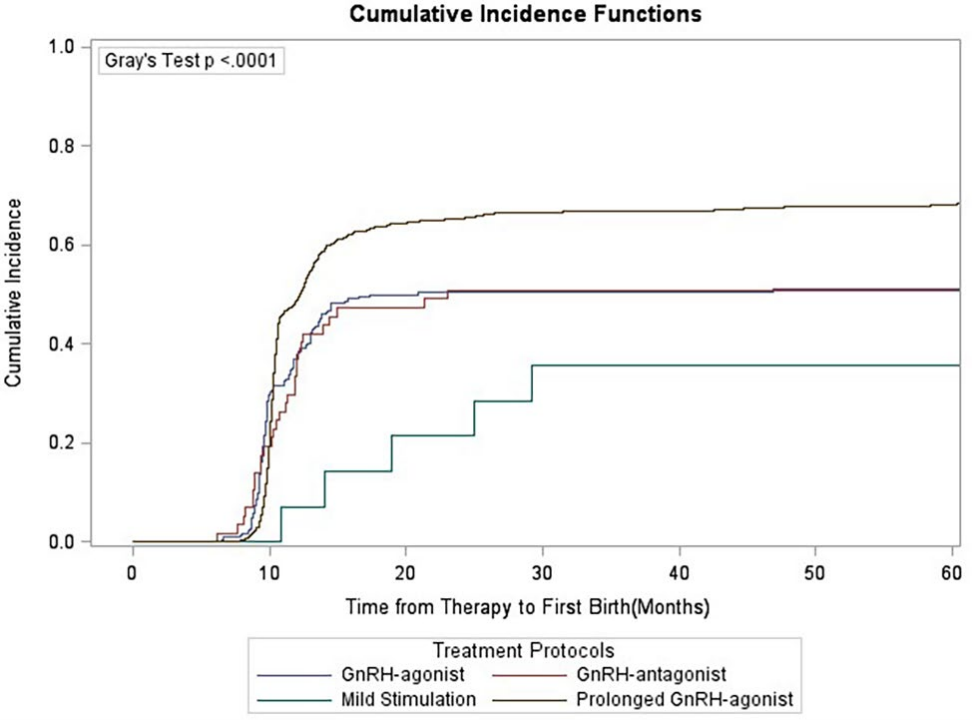
CLBRs, stratified for treatment protocols in four groups Prolonged GnRH-agonist, GnRH-agonist, GnRH-antagonist and Mild Stimulation. The distribution differed significantly in the groups (P < 0.0001)

Competition analysis showed that duration of infertility, primary/secondary type of infertility, stimulation protocols, starting dose of gonadotrophins and oocyte retrieved numbers were correlated with CLBR, and the difference was statistically significant (Figs. 1, 2, 3, 4, 5, 6 and 7). The older the female is, the lower CLBR tends to be, but the difference is not statistically significant (Fig. 2).

### Cox proportional risk regression model analysis

The Cox proportional risk regression model of PCOS patients showed that stimulation protocols had a significant impact on CLBR (Table 3). Women in the GnRH-antagonist protocol group and the mild stimulation protocol had lower CLBR than those in the Prolonged GnRH-agonist protocol [adjusted risk ratio (aHR): 0.71; 95% CI: 0.48–1.06; *p* = 0.0094 and aHR: 0.4; 95% CI: 0.15–1.08; *p* = 0.0001], which was statistically significant. PCOS patients with the starting dose of gonadotrophins greater than 112.5u had lower CLBR than those with less than 100u (aHR: 0.9; 95% CI: 0.64–1.27; *p* = 0.0358), which was statistically significant. There was a higher CLBR in women with the starting dose of gonadotrophins of 100u or 112.5u than in women with the starting dose of gonadotrophins of less than 100u, but there was no statistical significance. Women with 11–15 oocytes and 16–20 oocytes had higher CLBR than women with 1–9 oocytes (AHR: 1.54; 95% CI: 1.25–1.89; *p* < 0.0001 and AHR: 1.28; 95% CI: 1.04–1.58; *p* = 0.02), which was statistically significant. There was no significant difference in female age, duration of infertility and primary/secondary types of infertility in the model.

**Table 3:** Crude and aHR for cumulative live birth rate (CLBR) of women with PCOS

Independent covariates	Covariate strata	Crude hazard ratio (95% CI)	*p*-value	aHR (95% CI)	*p*-value
AGE(Years)	< 30				
	30–34	0.89(0.75–1.05)	0.175	0.92(0.77–1.1)	0.3541
	35–38	0.79(0.51–1.21)	0.2775	0.93(0.59–1.47)	0.7607
	> 38	0.52(0.17–1.61)	0.2537	0.72(0.23–2.25)	0.5674
Duration of Infertility (Years)	0~2				
	3~4	0.94(0.79–1.12)	0.48	0.88(0.73–1.05)	0.1029
	> 4	0.8(0.67–0.96)	0.015	0.78(0.64–0.94)	0.0895
Type of Infertility	Primary Infertility			,	
	Secondary Infertility	0.86(0.75-1)	0.0459	0.85(0.72–0.99)	0.5403
Treatment Protocols	Prolonged GnRH-agonist				
	GnRH-agonist	0.7(0.57–0.85)	0.0004	0.63(0.5–0.8)	0.1598
	GnRH-antagonist	0.67(0.46–0.97)	0.0356	0.71(0.48–1.06)	0.0094
	Mild Stimulation	0.35(0.14–0.83)	0.0175	0.4(0.15–1.08)	0.0001
Start Dose of Gonadotrophins(IU)	< 100				
	100	1.2(0.94–1.54)	0.1362	1.24(0.96–1.59)	0.0968
	112.5	1.27(1-1.62)	0.0497	1.24(0.97–1.6)	0.0698
	> 112.5	0.78(0.56–1.08)	0.1278	0.9(0.64–1.27)	0.0358
Oocytes Retrieved	1~10				
	11~15	1.54(1.26–1.88)	< 0.0001	1.54(1.25–1.89)	< 0.0001
	16~20	1.29(1.05–1.58)	0.0151	1.28(1.04–1.58)	0.0221
	> 20	0.95(0.77–1.17)	0.6203	0.95(0.76–1.18)	0.6249

## Discussions

Since the first live birth of a thawed frozen embryo in 1984, there has been an increase in the number of thawed frozen embryo transfers and the associated pregnancy rate due to advancements in technology [15]. This trend is supported by the implementation of the strategy of single embryo transfer and the prevention of moderate to OHSS in high-risk women [16]. Consequently, relying solely on the single cycle live birth rate is insufficient for evaluating the success rate of IVF patients. The concept of CLBR has emerged as a method to comprehensively assess the IVF success rate for both fresh and frozen embryo transfers [17]. Patients with PCOS, who are highly reactive and prone to OHSS, should not solely rely on the success rate of IVF based on fresh embryo transfer. A comprehensive report should include not only the results of fresh embryo transfer but also the outcomes of frozen-thawed embryo transfer in order to provide a more comprehensive success rate [12]. Therefore, the CLBR is of great importance in predicting the prognosis of PCOS patients undergoing IVF/ICSI-ET.

In our retrospective analysis of data from a single-center, we included a total of 1380 PCOS patients who underwent the 1st oocyte retrieval cycle. The study monitored birth outcomes over a period of 2–4 years, encompassing both fresh and thawed cycles. The conservative cumulative live birth rate was determined to be 63.48%. Upon analyzing the data from the cumulative live birth group (Group A) and the non-cumulative live birth group (Group B), statistically significant differences were observed in various factors, including duration of infertility, primary/secondary type of infertility, stimulation protocols, starting dose of gonadotrophins, oocyte retrieved, and the number of embryos available. Taking these factors and female age into Cox proportional risk regression model, we found that the stimulation protocols, starting dose of gonadotrophins and oocyte retrieved had significant effects on CLBR.

The stimulation protocols employed during IVF treatment have a significant impact on the oocyte, embryo, and endometrium of patients with PCOS, consequently influencing the success rate of the treatment. In our research, we observed that women in the GnRH-antagonist protocol group and those undergoing mild stimulation protocol exhibited lower CLBR compared to those following the Prolonged GnRH-agonist protocol, and this difference was statistically significant. Our study encompassed four ovulation induction therapies for PCOS patients, namely the Prolonged GnRH-agonist protocol, GnRH-agonist protocol, GnRH-antagonist protocol, and mild stimulation protocol. The standard GnRH-agonist protocol represents the conventional approach in this context, which is most widely used in the early IVF-ET treatment of PCOS patients [18]. The prolonged GnRH-agonist protocol, GnRH-antagonist protocol, and mild stimulation protocol are all variations of the stimulation protocol being compared. The prolonged GnRH-agonist protocol, specifically, has been found to increase down-regulation time and is commonly used in IVF-ET treatment for endometriosis [19]. In the treatment of PCOS, the prolonged GnRH-agonist protocol has been shown to enhance endometrial receptivity by extending the down-regulating time, resulting in higher rates of implantation and pregnancy, without an increase in the occurrence of OHSS [20]. Meanwhile, it is worth noting that while the GnRH-antagonist protocol has demonstrated a reduced incidence of OHSS, it often exhibits a lower rate of successful pregnancies [21]. In contrast, the treatment of PCOS patients using a mild stimulation protocol, similar to the antagonist program but without down-regulation and with lower doses of ovulation-promoting drugs, has shown comparable outcomes. This alternative approach, when compared to the standard GnRH-agonist protocol, has been associated with a decreased OHSS rate but a higher rate of cycle cancellations. However, the CLBR following repeated ovulation remains unaffected and is primarily employed to address declining ovarian function. [22–24]. In summary, the Prolonged GnRH-agonist protocol improves the CLBR of 1st oocyte collection in PCOS patients, and is the recommended scheme for IVF treatment in PCOS patients.

Due to the presence of abnormal endocrine levels, individuals with PCOS often exhibit elevated androgen levels, impaired glucose tolerance, and insulin resistance [25]. In the latest study by Zijiang Chen’s team, they showed that in infertile women with PCOS, frozen embryo transfer was associated with a higher rate of live birth and a lower risk of ovarian hyperstimulation syndrome compared to fresh embryo transfer [26]. To mitigate the occurrence of OHSS, the initial dosage of gonadotropins administered to PCOS patients holds significant importance. The recommended approach involves implementing a low-dose follicle-stimulating hormone (FSH) stimulation strategy [7, 8]. Typically, the dosage of commonly employed ovulation-inducing medications ranges from 75-112.5u. Our statistical analysis also supports the finding that patients with PCOS who received a starting dose of gonadotrophins greater than 112.5u had a lower CLBR compared to those who received less than 100u. However, it is worth noting that although not statistically significant, women who received a starting dose of gonadotrophins of 100u or 112.5u had a higher CLBR than those who received less than 100u. This observation may be attributed to the fact that outcome parameters tend to increase with the degree of response to gonadotrophin stimulation, reach a plateau, and then decrease with a stronger response to stimulation. 2) Lower doses of gonadotrophins have been found to result in smaller reactions, which may be beneficial for more sensitive patients, such as those with PCOS [7].

A recent clinical randomized controlled study, comparing the outcomes of GnRH-antagonist and GnRH-agonist protocols in patients without PCOS, demonstrated that the number of oocytes retrieved significantly influenced the CLBR in both protocols. Furthermore, the subgroup with 1–3 oocytes retrieved exhibited the lowest cumulative live birth rate (CLBR), while the subgroup with > 15 oocytes retrieved exhibited the highest CLBR. However, no significant difference was observed in the CLBR between the GnRH-antagonist and GnRH-agonist protocols when comparing each oocyte retrieved subgroup. These findings suggest that the impact of the number of oocytes retrieved on CLBR is not influenced by the choice of protocol [27]. Previous studies have also reported that the optimal number of oocytes retrieved for achieving a good CLBR and preventing severe ovarian hyperstimulation syndrome (OHSS) in non-PCOS patients undergoing GnRH-antagonist protocols is between 6 and 15 [9]. However, a separate study examining women aged 35–40 found that the optimal number of oocytes retrieved was between 10 and 14 [28]. Additionally, a retrospective analysis of IVF-ET treatment in patients with polycystic ovary syndrome (PCOS) revealed a significant positive correlation between the number of oocytes retrieved and clinical live birth rate (CLBR). It is worth noting that while retrieving more than 10 oocytes did not result in a significant improvement in CLBR, it did lead to the production of surplus embryos [11]. Consistent with previous research, our findings indicate that women with PCOS who had between 11 and 15, as well as 16 and 20, oocytes retrieved had higher CLBR compared to those with 1–9 oocytes in IVF-ET treatment.

Furthermore, a study has indicated that the classification of PCOS is closely associated with the CLBR, particularly in patients with type D PCOS, characterized by normal androgen levels, where the CLBR reaches 48%, surpassing that of other PCOS patients with a high androgen phenotype [29]. Another study has demonstrated that patients with type D PCOS also exhibit a higher clinical pregnancy rate compared to other patient groups [30]. Consequently, it can be inferred that heightened reactivity may serve as a pivotal factor contributing to a slightly increased optimal number of oocytes retrieved in PCOS patients.

## Conclusion

Patients with PCOS represent a challenge for reproductive medicine. According to our statistical results, the CLBR of PCOS patients increased significantly after a 1st oocyte collection when we used prolonged GnRH-agonist protocol, when the first starting dose of gonadotrophins was 100u-112.5u and when the number of oocytes obtained was 11–15 and 16–20.

## Data Availability

All data generated or analyzed during this study are included in this published article [and its supplementary information files].

## References

[1] Balen AH (2016). The management of anovulatory infertility in women with polycystic ovary syndrome: an analysis of the evidence to support the development of global WHO guidance. Hum Reprod Update.

[2] Melo AS, Ferriani RA, Navarro PA (2015). Treatment of infertility in women with polycystic ovary syndrome: approach to clinical practice. Clin (Sao Paulo).

[3] Sha T (2019). A meta-analysis of pregnancy-related outcomes and complications in women with polycystic ovary syndrome undergoing IVF. Reprod Biomed Online.

[4] Yan J (2012). Effect of maternal age on the outcomes of in vitro fertilization and embryo transfer (IVF-ET). Sci China Life Sci.

[5] Ding W (2019). Impact of female obesity on cumulative live birth rates in the First Complete ovarian stimulation cycle. Front Endocrinol (Lausanne).

[6] Chen Y, Zhao J, Zhang H (2018). Comparative effectiveness of three ovarian hyperstimulation protocol in in vitro fertilization (IVF) cycles for women with polycystic ovary syndrome. Med Sci Monit.

[7] Fischer D (2016). Avoiding OHSS: controlled ovarian low-dose stimulation in women with PCOS. Geburtshilfe Frauenheilkd.

[8] Oudshoorn SC (2017). Individualized versus standard FSH dosing in women starting IVF/ICSI: an RCT. Part 2: the predicted hyper responder. Hum Reprod.

[9] Ji J (2013). The optimum number of oocytes in IVF treatment: an analysis of 2455 cycles in China. Hum Reprod.

[10] Casano S (2012). MILD ovarian stimulation with GnRH-antagonist vs. long protocol with low dose FSH for non-PCO high responders undergoing IVF: a prospective, randomized study including thawing cycles. J Assist Reprod Genet.

[11] Chen YH (2017). Cumulative live birth and surplus embryo incidence after frozen-thaw cycles in PCOS: how many oocytes do we need?. J Assist Reprod Genet.

[12] Chen ZJ (2016). Fresh versus frozen embryos for infertility in the polycystic ovary syndrome. N Engl J Med.

[13] Maheshwari A, McLernon D, Bhattacharya S (2015). Cumulative live birth rate: time for a consensus?. Hum Reprod.

[14] Rotterdam E (2004). Revised 2003 consensus on diagnostic criteria and long-term health risks related to polycystic ovary syndrome. Fertil Steril.

[15] Xing W, Ou J, Cai L (2018). Thawed embryo transfer and ectopic pregnancy: a meta-analysis. Arch Gynecol Obstet.

[16] van Loendersloot LL (2017). Cost-effectiveness of single versus double embryo transfer in IVF in relation to female age. Eur J Obstet Gynecol Reprod Biol.

[17] Germond M (2004). What is the most relevant standard of success in assisted reproduction? The cumulated singleton/twin delivery rates per oocyte pick-up: the CUSIDERA and CUTWIDERA. Hum Reprod.

[18] Teede HJ (2018). Recommendations from the international evidence-based guideline for the assessment and management of polycystic ovary syndrome. Fertil Steril.

[19] Maged AM (2018). Effect of prolonged GnRH agonist downregulation on ICSI outcome in patients with endometriomas of Less Than 5 cm: a Randomized Controlled Trial. Reprod Sci.

[20] Gong F (2015). A modified ultra-long pituitary downregulation protocol improved endometrial receptivity and clinical outcome for infertile patients with polycystic ovarian syndrome. Exp Ther Med.

[21] Lambalk CB (2017). GnRH antagonist versus long agonist protocols in IVF: a systematic review and meta-analysis accounting for patient type. Hum Reprod Update.

[22] D’Amato G (2018). Mild ovarian stimulation with letrozole plus fixed dose human menopausal gonadotropin prior to IVF/ICSI for infertile non-obese women with polycystic ovarian syndrome being pre-treated with metformin: a pilot study. Reprod Biol Endocrinol.

[23] Nargund G, Datta AK, Fauser B (2017). Mild stimulation for in vitro fertilization. Fertil Steril.

[24] Tshzmachyan R, Hambartsoumian E (2020). The role of Letrozole (LE) in controlled ovarian stimulation (COS) in patients at high risk to develop ovarian hyper stimulation syndrome (OHSS). A prospective randomized controlled pilot study. J Gynecol Obstet Hum Reprod.

[25] Bednarska S, Siejka A (2017). The pathogenesis and treatment of polycystic ovary syndrome: what’s new?. Adv Clin Exp Med.

[26] Chen Z-J et al. *Fresh versus frozen embryos for infertility in the polycystic ovary syndrome*. N Engl J Med. 2016.375(6):p. 523 – 33. 10.1056/NEJMoa1513873 27509101

[27] Toftager M (2017). Cumulative live birth rates after one ART cycle including all subsequent frozen-thaw cycles in 1050 women: secondary outcome of an RCT comparing GnRH-antagonist and GnRH-agonist protocols. Hum Reprod.

[28] Zhou J (2017). Association between the number of oocytes retrieved and cumulative live birth rate in women aged 35–40 years undergoing long GnRH agonist IVF/ICSI cycles. Arch Gynecol Obstet.

[29] Ramezanali F (2016). Assisted reproductive outcomes in women with different polycystic ovary syndrome phenotypes: the predictive value of anti-Müllerian hormone. Reprod Biomed Online.

[30] De Vos M (2018). Cumulative live birth rates after IVF in patients with polycystic ovaries: phenotype matters. Reprod Biomed Online.

